# Cortical inhibitory markers of lifetime suicidal behavior in depressed adolescents

**DOI:** 10.1038/s41386-018-0040-x

**Published:** 2018-03-14

**Authors:** Charles P. Lewis, Paul A. Nakonezny, Caren J. Blacker, Jennifer L. Vande Voort, John D. Port, Gregory A. Worrell, Hang Joon Jo, Zafiris J. Daskalakis, Paul E. Croarkin

**Affiliations:** 1 Department of Psychiatry and Psychology, Mayo Clinic, Rochester, MN USA; 2 Department of Psychiatry, University of Texas Southwestern Medical Center, Dallas, TX USA; 3 Department of Clinical Sciences, Division of Biostatistics, University of Texas Southwestern Medical Center, Dallas, TX USA; 4 Department of Radiology, Mayo Clinic, Rochester, MN USA; 5 Department of Neurology, Mayo Clinic, Rochester, MN USA; 6 Department of Neurologic Surgery, Neural Engineering Lab, Mayo Clinic, Rochester, MN USA; 7 Department of Psychiatry, Faculty of Medicine, University of Toronto, Toronto, ON Canada; 8 Temerty Centre for Therapeutic Brain Intervention, Campbell Family Mental Health Research Institute, Centre for Addiction and Mental Health, University of Toronto, Toronto, ON Canada

## Abstract

Although suicide is the second-leading cause of death in adolescents and young adults worldwide, little progress has been made in developing reliable biological markers of suicide risk and suicidal behavior. Converging evidence suggests that excitatory and inhibitory cortical processes mediated by the neurotransmitters glutamate and γ-aminobutyric acid (GABA) are dysregulated in suicidal individuals. This study utilized single- and paired-pulse transcranial magnetic stimulation (TMS) to assess excitatory and inhibitory cortical functioning in healthy control adolescents (*n* = 20), depressed adolescents without any history of suicidal behavior (“Depressed”, *n* = 37), and depressed adolescents with lifetime history of suicidal behavior (“Depressed+SB”, *n* = 17). In a fixed-effects general linear model analysis, with age, sex, and depression severity as covariates, no significant group main effects emerged for resting motor threshold, intracortical facilitation, short-interval intracortical inhibition, or cortical silent period. However, group main effects were significant for long-interval intracortical inhibition (LICI) at interstimulus intervals (ISIs) of 100 ms and 150 ms, but not 200 ms. Depressed+SB adolescents demonstrated impaired LICI compared to healthy control and Depressed adolescents, while healthy control and Depressed participants did not differ in LICI. Multiple linear robust regression revealed significant positive linear relationships between lifetime suicidal behavior severity and impairment in LICI at 100-ms and 150-ms ISIs. In a post hoc receiver operating characteristic analysis, LICI significantly discriminated Depressed from Depressed+SB youth in 100-ms and 150-ms paradigms. These findings suggest that GABA_B_ receptor-mediated inhibition is distinctly dysregulated in depressed adolescents with histories of suicidal behavior. Further research is warranted to establish the utility of cortical inhibition in the assessment of suicide risk and as a target for treatment interventions.

## Introduction

Suicide is the second most common cause of death among adolescents and young adults in the United States [1] and globally [2], with 17.7% of U.S. adolescents reporting having seriously considered suicide, 14.6% having made a suicide plan, and 8.6% having attempted suicide within the preceding 12 months [3]. Additionally, suicidal ideation and attempts during childhood and adolescence predict later suicidal behavior in adulthood [4]. Notably, rates of suicide attempts among adults have increased in recent years [5]. Despite the immense personal, societal, and healthcare burdens associated with suicidal behavior in young people, assessing suicide risk remains a challenge in clinical practice, frequently complicated by the lack of objective, verifiable data. The predictive value of identified suicide risk factors, however, is limited and has not improved substantially over time, despite decades of research [6]. Furthermore, biological correlates investigated to date are weak predictors of suicidal behavior [7]. There is growing recognition of the need for additional quantifiable neurobiological markers to augment current clinical assessments in order to more reliably identify and monitor those at high risk for suicide [8, 9].

Previous research has implicated the hypothalamic–pituitary–adrenal stress response system and serotonergic and noradrenergic synaptic transmission in suicide [8] and suicidal behavior [9]. However, increasing evidence suggests that suicidal individuals also have aberrances in amino acid neurotransmitter systems, including γ-aminobutyric acid (GABA) and glutamate, respectively the principal inhibitory and excitatory transmitters of the mammalian brain. Data supporting the involvement of GABA and glutamate in the pathophysiology of suicidal behavior come from diverse methodologies, including gene association studies [10], analyses of postmortem brain gene expression [11–14] and receptor binding [15, 16], immunohistochemical studies [17], epigenetic research [18], and cerebrospinal fluid studies [19, 20].

The physiology of cortical GABAergic and glutamatergic systems can be probed noninvasively with single- and paired-pulse transcranial magnetic stimulation (TMS) techniques. Stimulation of the motor cortex while varying the intensity of the magnetic pulse and the interstimulus interval (for paired-pulse paradigms) elicits distinct excitatory and inhibitory effects that can be measured with simultaneous electromyographic (EMG) recording of the contralateral limb. These include inhibitory effects mediated by ionotropic GABA_A_ and metabotropic GABA_B_ receptors, such as the cortical silent period (CSP), short-interval intracortical inhibition (SICI), and long-interval intracortical inhibition (LICI), as well as indices of glutamate receptor-mediated excitatory functioning, such as the resting motor threshold (RMT) and intracortical facilitation (ICF). Mechanisms of these excitatory and inhibitory TMS measures have been elucidated through extensive animal and human experiments (for a review, see ref. [21]).

Single- and paired-pulse TMS have been used to investigate cortical excitability and inhibition in a variety of psychiatric conditions, including depression in adult populations [22, 23]. Meta-analysis of adult studies [24] found shorter CSP and reduced SICI in depressed individuals compared to healthy controls. However, adolescents with major depressive disorder appear to have a different pattern of altered cortical excitability [25]. Although suicidal ideation and behavior are common symptoms among adolescents with depression, it remains undetermined whether suicidality confers a neurobiological signature distinct from that of depression alone. To our knowledge, no prior research has utilized TMS to investigate cortical excitability and inhibition as an index of suicidality. Thus, the primary aim of this study was to examine single- and paired-pulse TMS measures of cortical excitability (RMT, ICF) and inhibition (CSP, SICI, LICI) in depressed adolescents with and without histories of suicidal behavior and in a healthy control comparator group. We hypothesized that adolescents with histories of suicidal behavior would have impaired inhibition (CSP, SICI, LICI) compared to healthy control and depressed adolescents without histories of suicidal behavior. As a secondary aim, we examined the relationship between severity of lifetime suicidal behavior and TMS measures of cortical inhibition (CSP, SICI, LICI) and excitability (RMT, ICF). Additionally, as an exploratory analysis, we examined the ability of a cortical inhibitory measure (LICI) to discriminate depressed youth without histories of suicidal behavior from those with lifetime suicidal behavior.

## Materials and methods

The current study is based on a pooled sample from three extant individual studies that utilized identical clinical assessments and TMS methods. In this cross-sectional study, a clinical assessment preceded TMS cortical excitability and inhibition measures, which were obtained during a single session. The study was approved by the institutional review boards of Mayo Clinic (Rochester, MN, USA) and the University of Texas Southwestern Medical Center (Dallas, TX, USA).

### Participants

Participants consisted of treatment-seeking depressed adolescents recruited from clinical practice and healthy adolescents recruited through community advertising. Prior to any study assessments or procedures, all minor participants provided written assent, and a parent or guardian granted written informed consent; all participants aged ≥18 years provided written informed consent.

Eligible participants were English-speaking adolescents between the ages of 12 and 21 years; minor participants also were required to have a parent or guardian fluent in English. Participants underwent a structured diagnostic interview, the Schedule for Affective Disorders and Schizophrenia for School-Age Children (K-SADS-PL; [26]). Participants eligible for the Healthy Control group (*n* = 20) did not meet criteria for any psychiatric diagnosis (past or present), had no family history of psychiatric diagnosis in first-degree relatives, and had no history of psychotropic medication use, psychotherapy, or suicidal behavior. Eligibility for the depressed groups required the diagnosis of a depressive disorder on the K-SADS-PL. Depression severity was assessed on the clinician-rated Children’s Depression Rating Scale, Revised (CDRS-R; [27]); in the interest of broad-based external validity, and considering that youth with a range of depressive symptoms may engage in suicidal behavior, no minimal score was required. Depressed participants’ recent and lifetime histories of suicidal ideation and behavior were assessed on clinical interview using the Columbia–Suicide Severity Rating Scale (C-SSRS; [28]). Participants with either no history of suicidal behavior or a history of isolated non-suicidal self-injurious behavior (i.e., self-injury with clearly non-suicidal intent and no history of suicidal preparatory behavior, aborted or interrupted attempt, or actual attempt) were classified in the depressed without lifetime history of suicidal behavior (“Depressed”) group (*n* = 37). Participants with any lifetime history of preparatory behavior, aborted or interrupted attempt, or actual suicide attempt were classified in the depressed with lifetime history of suicidal behavior (“Depressed+SB”) group (*n* = 17). Classification was also confirmed based on review of clinical records and consultation with the treating psychiatrist. All structured interviews and clinical assessments were performed by a board-certified child and adolescent psychiatrist (PEC).

Exclusion criteria included increased risk of seizure (e.g., personal history of epilepsy, febrile or unprovoked seizures, intracranial tumor, or intracranial surgical procedure; family history of epilepsy) or other contraindication to TMS (implanted ferromagnetic device or metallic fragments, not including orthodontic hardware) as assessed on the TMS Adult Safety Screen [29]. Additionally, adolescents were excluded if they had primary psychiatric diagnoses other than depressive disorders, were pregnant or at risk of pregnancy (for post-menarchal female participants), had unstable medical conditions, or were evaluated by a study psychiatrist to be at imminent risk of medically serious self-injury or suicide at the time of participation.

### Procedures and measures

All study participants underwent single- and paired-pulse TMS of the left primary motor cortex to assess cortical excitability and inhibition. During stimulation, participants remained seated and wore earplugs for noise safety and comfort. Electromyographic (EMG) electrodes affixed to the surface of the skin overlying the contralateral (right) abductor pollicis brevis (APB) muscle recorded motor evoked potentials (MEPs) throughout the TMS session. Procedures for determining coil position and excitatory and inhibitory measures have been published previously [30]. Participants were permitted to continue their prescribed psychotropic medications during the study, although stimulants were held on the day of TMS testing.

Magnetic pulses were generated by two Magstim 200 stimulators connected by a BiStim module (Magstim Co. Ltd., Whitland, Wales, UK) and delivered with a figure-of-eight electromagnetic coil (70 mm diameter, each loop) held tangentially against the scalp. The area of motor cortex responsible for APB movement was localized by stimulating with single pulses at a constant intensity while shifting the coil in 1-cm increments until maximal muscle movement was observed visually. After the optimal APB position was localized, coil position was held constant while the stimulus intensity was increased gradually. The stimulus intensity at which MEP amplitude observed on EMG was at least 50 μV in 5 out of 10 trials was defined as the resting motor threshold (RMT) [31].

The CSP was determined by single-pulse stimulation of the left primary motor cortex at 140% of the RMT while the participant voluntarily contracted the right APB at 20% of maximum contraction (determined by hand-held dynamometer). The duration of interruption in voluntary motor activity (from the beginning of the TMS pulse to the resumption of motor activity on EMG) was measured in 10 trials and averaged.

**Fig. 1:**
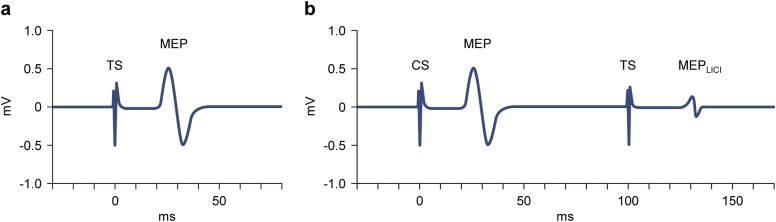
Schematic illustration of typical electromyographic recordings of single- and paired-pulse transcranial magnetic stimulation of the primary motor cortex as measured in electrodes overlying the abductor pollicis brevis (APB). **a** When a single magnetic test stimulus (TS) at an intensity above the resting motor threshold is applied to the area of the primary motor cortex corresponding to the APB (at time = 0), a motor evoked potential (MEP) is recorded in the contralateral APB electrode shortly afterward. **b** In the long-interval intracortical inhibition (LICI) paired-pulse paradigm, a suprathreshold (i.e., above the resting motor threshold) conditioning stimulus (CS) is delivered to the primary motor cortex (at time = 0), which results in an unconditioned MEP. After an interstimulus interval of 100–200 ms (100 ms shown), a second, identical test stimulus (TS) is administered. The resulting conditioned MEP (MEP_LICI_) is diminished in amplitude. This inhibition is mediated predominantly by cortical GABA_B_ receptors.

For SICI, LICI, and ICF measures, paired conditioning and test stimuli were delivered while the APB was at rest. In the SICI and ICF paradigms, a subthreshold (80% RMT) conditioning stimulus preceded the test stimulus, whose intensity was calibrated to generate peak-to-peak MEP amplitudes of 1.0 mV. A schematic representation of an EMG recording of an MEP resulting from a single TMS test stimulus is illustrated in Fig. 1a. For SICI, interstimulus intervals (ISIs) were 2 ms and 4 ms, while for the ICF paradigm, ISIs were 10 ms, 15 ms, and 20 ms. By contrast, the LICI paradigm (Fig. 1b) involved suprathreshold conditioning and test stimuli (both calibrated to result in 1.0-mV peak-to-peak MEP amplitudes) and ISIs of 100 ms, 150 ms, and 200 ms. Amplitudes of the conditioned MEPs (the MEPs resulting from the test stimuli) were recorded on EMG (12 trials at each ISI for SICI and ICF; 10 trials at each ISI for LICI) and averaged. For all paired-pulse measures, amplitudes of conditioned MEPs are expressed as ratios to the mean unconditioned MEP amplitude. Trials of all three paired-pulse paradigms were performed concurrently in a randomized and counterbalanced order.

### Outcome variables

The primary outcomes were TMS measures of cortical inhibition (CSP duration; SICI and LICI conditioned/unconditioned MEP amplitude ratios) and excitability (RMT; ICF conditioned/unconditioned MEP amplitude ratios).

### Independent variables and covariates

The primary independent variable was group membership (Healthy Control vs. Depressed vs. Depressed+SB). Participants’ highest level of lifetime suicidal behavior severity, as assessed on the C-SSRS at study entry, was a predictor (independent) variable in the regression models. An ordinal scale of suicidal behavior was operationally defined as: 0 = none; 1 = non-suicidal self-injury; 2 = planning/preparation for a suicide attempt; 3 = interrupted or aborted attempt; 4 = suicide attempt. Sex, age in years, and depressive symptom severity at study entry were included as covariates in the models. Depression severity was assessed using the CDRS-R total score.

### Statistical analysis

Demographic and clinical characteristics of the participants were described using the sample mean and standard deviation for continuous variables and the frequency and percentage for categorical variables. One-way analysis of variance (for continuous variables) and Fisher’s exact test (for categorical variables) were used to test for differences among the three groups (Healthy Control vs. Depressed vs. Depressed+SB) on demographic characteristics and between the two depressed groups (Depressed vs. Depressed+SB) on clinical characteristics.

For the primary aim, a fixed-effects general linear model (GLM) was used to examine the group main effect (Healthy Control vs. Depressed vs. Depressed+SB) on each of the TMS outcome measures of cortical inhibition (CSP, SICI, LICI) and excitability (RMT, ICF). Group least squares means of the TMS outcome measure estimates were compared for a total of three post hoc pair-wise contrasts, with *p*-values adjusted for multiple comparisons using the Tukey–Kramer method. A weight statement was included in the model to account for (and down-weight) any potential outliers (see supplementary materials and methods for details). For the secondary aim, multiple linear robust regression (with MM estimation) and the Spearman partial correlation coefficient (*r*_s_) were used to examine the relationship between severity of lifetime suicidal behavior and each TMS outcome measure, while adjusting for age, sex, and CDRS-R total score. Additional technical details regarding the GLM and robust regression analyses are available in supplementary materials and methods.

Finally, as a post hoc sensitivity analysis, a receiver operating characteristic (ROC) analysis alongside the area under the curve (AUC) was conducted to determine the optimal conditioned/unconditioned MEP amplitude ratio cutpoints for each of the LICI paradigms (100-ms, 150-ms, and 200-ms ISIs), based on the Youden index, in discriminating Depressed vs. Depressed+SB status. The AUC associated with each optimal cutpoint was tested against a nominal area of 0.50 using the *Z* statistic. The 95% binomial exact confidence intervals (CIs) were calculated for the AUC. Sensitivity, specificity, positive predictive value (PPV), and negative predictive value (NPV) were reported for each of the optimal cutpoints.

Statistical analyses were carried out using the SAS software, version 9.4 (SAS Institute, Inc., Cary, NC, USA). The procedures of PROC GLIMMIX and PROC ROBUSTREG in the SAS software were used to conduct the analyses for the primary and secondary aims. The level of significance was set at *α* = 0.05 (two-tailed), and to address multiple testing (where applicable), *p*-values were adjusted using the False Discovery Rate (FDR) procedure [32].

## Results

### Participant characteristics

Of the entire sample of 74 adolescent participants, 59.46% were female and 67.56% were non-Hispanic Caucasian. The mean age was 15.33 ± 1.89 years (range = 12-20 years). Mean CDRS-R total score was 36.59 ± 15.78. Of the 74 youths, 66.22% reported no lifetime history of suicidal behavior, but 10.81% reported non-suicidal self-injury. Demographic and clinical characteristics of the overall sample and each group are shown in Table 1, with comparisons of all three groups on demographic characteristics and of the two depressed groups on clinical characteristics. A list of concurrent psychotropic medications taken by participants in the Depressed and Depressed+SB groups at the time of TMS testing is provided in Supplementary Table S1.

**Table 1:** Demographic and clinical characteristics of the overall sample and by group

Participant characteristic	Overall sample (*n* = 74)	Healthy Control (*n* = 20)	Depressed^a^ (*n* = 37)	Depressed+SB^b^ (*n* = 17)	*p*-value (*p*_FDR_)
Demographic					
Age in years, M ± SD	15.33 ± 1.89	14.20 ± 1.76	15.70 ± 1.88	15.88 ± 1.57	0.005 (0.015)
Sex, % (*n*)					0.319 (0.362)
Female	59.46% (44)	45.00% (9)	64.86% (24)	64.71% (11)	
Male	40.54% (30)	55.00% (11)	35.14% (13)	35.29% (6)	
Right-handed, % (*n*)	93.24% (69)	90.00% (18)	100% (37)	82.35% (14)	0.023 (0.051)
Race/ethnicity, % (*n*)					0.001 (0.004)
African-American	16.22% (12)	45.00% (9)	5.41% (2)	5.88% (1)	
Asian-American	2.70% (2)	0.00% (0)	5.41% (2)	0.00% (0)	
Caucasian, Non-Hispanic	67.56% (50)	40.00% (8)	78.37% (29)	76.47% (13)	
Hispanic/Latino	2.70% (2)	5.00% (1)	2.70% (1)	0.00% (0)	
Native American	1.35% (1)	0.00% (0)	2.70% (1)	0.00% (0)	
Multiethnic/other	9.46% (7)	10.00% (2)	5.41% (2)	17.65% (3)	
Clinical					
CDRS-R total score, M ± SD	36.59 ± 15.78	19.65 ± 1.69	41.48 ± 13.03	45.88 ± 15.64	0.285 (0.362)
Length of illness (years), M ± SD	2.27 ± 1.96	N/A	2.08 ± 1.93	2.67 ± 2.05	0.313 (0.362)
Currently prescribed psychotropic medications, % (*n*)	43.24% (32)	0.00% (0)	54.05% (20)	70.59% (12)	0.372 (0.372)
Familial psychiatric history, % (*n*)	69.86% (51)	0.00% (0)	94.44% (34)^c^	100% (17)	0.322 (0.362)
Maximal lifetime history of suicidal behavior, % (*n*)					0.0001 (0.001)
None	66.22% (49)	100% (20)	78.38% (29)	0.00% (0)	
Non-suicidal self-injury	10.81% (8)	0.00% (0)	21.62% (8)	0.00% (0)	
Planning/preparation for a suicide attempt	2.70% (2)	0.00% (0)	0.00% (0)	11.76% (2)	
Interrupted or aborted attempt	6.76% (5)	0.00% (0)	0.00% (0)	29.41% (5)	
Suicide attempt	13.51% (10)	0.00% (0)	0.00% (0)	58.82% (10)	

Note: The means (M) presented in this table are the sample means; SD = standard deviation; N/A = not applicable. To identify differences among the characteristics of the groups, one-way ANOVA (for continuous variables) and Fisher’s exact test (for categorical variables) were used. We tested for differences among the three groups on the demographic characteristics and for differences between the two depressed groups on the clinical characteristics. Correction for multiple comparisons was performed using the False Discovery Rate (FDR) method described by Benjamini and Hochberg [32].

^a^Depressive disorder without lifetime history of suicidal behavior (SB)

^b^Depressive disorder with lifetime history of SB

^c^1 missing observation

### Cortical excitability

The GLM analysis revealed no significant group main effect for RMT (*F*_2,68_ = 0.16, *p* = 0.8484, *p*_FDR_ = 0.8484). For the ICF paradigm, the GLM analysis showed no significant group main effects for conditioned/unconditioned MEP amplitude ratio at the 10-ms ISI (*F*_2,67_ = 1.04, *p* = 0.3601, *p*_FDR_ = 0.4801), 15-ms ISI (*F*_2,67_ = 1.18, *p* = 0.3127, *p*_FDR_ = 0.4801), or 20-ms ISI (*F*_2,68_ = 0.0869, *p* = 0.0945, *p*_FDR_ = 0.3780). Least squares mean and standard error values for TMS measures of cortical excitability are reported in Table 2.

The multiple linear robust regression (Table 3), while adjusting for age, sex, and CDRS-R total score, revealed a non-significant negative linear relationship between lifetime suicidal behavior severity and RMT (*b̂* = -0.5160, *p* = 0.6333), as well as non-significant negative linear relationships between lifetime suicidal behavior severity and conditioned/unconditioned MEP amplitude ratio in the 10-ms (*b̂* = -0.0655, *p* = 0.1812), 15-ms (*b̂* = -0.0410, *p* = 0.3986), and 20-ms (*b̂* = -0.0483, *p* = 0.4059) ICF paradigms.

**Table 2:** Cortical excitability and inhibition results by group

TMS measure	Least squares mean (SE)			
	Healthy Control (*n* = 20)	Depressed^a^ (*n* = 37)	Depressed+SB^b^ (*n* = 17)	*p*-value^c^ (*p*_FDR_)
Cortical excitability				
RMT^d^	51.757 (3.193)	53.591 (1.607)	52.413 (2.738)	0.8484 (0.8484)
ICF^e^				
ISI = 10 ms	1.476 (0.128)	1.580 (0.086)	1.398 (0.102)	0.3601 (0.4801)
ISI = 15 ms	1.407 (0.132)	1.658 (0.078)	1.553 (0.128)	0.3127 (0.4801)
ISI = 20 ms	1.295 (0.147)	1.692 (0.087)	1.528 (0.138)	0.0945 (0.3780)
Cortical inhibition				
CSP^f^	0.184 (0.011)	0.166 (0.005)	0.150 (0.010)	0.1216 (0.2432)
SICI^e^				
ISI = 2 ms	0.505 (0.069)	0.430 (0.043)	0.406 (0.066)	0.6061 (0.6061)
ISI = 4 ms	0.751 (0.091)	0.642 (0.052)	0.707 (0.074)	0.5158 (0.6061)
LICI^e^				
ISI = 100 ms	0.122 (0.059)	0.249 (0.042)	0.530 (0.075)	0.0002 (0.0012)
ISI = 150 ms	0.151 (0.072)	0.351 (0.042)	0.623 (0.099)	0.0013 (0.0039)
ISI = 200 ms	0.975 (0.143)	0.795 (0.084)	0.713 (0.147)	0.5147 (0.6061)

Note: *p*_FDR_ calculated separately by cortical excitability vs. inhibition. Least squares means are adjusted for age, sex, and CDRS-R total score.

*RMT* resting motor threshold, *ICF* intracortical facilitation, *ISI* interstimulus interval, *CSP* cortical silent period, *SICI* short-interval intracortical inhibition, *LICI* long-interval intracortical inhibition, *SE* robust/empirical standard error estimate, *FDR* False Discovery Rate described by Benjamini and Hochberg [32]

^a^Depressive disorder without lifetime history of suicidal behavior (SB)

^b^Depressive disorder with lifetime history of SB

^c^Omnibus test of the group main effect (Healthy Controls vs. Depressed vs. Depressed+SB) on each of the TMS outcome measures

^d^RMT is expressed as the percentage of maximal device output

^e^ICF, SICI, and LICI conditioned motor evoked potential (MEP) amplitudes are expressed as ratios to the mean unconditioned MEP amplitudes

^f^CSP duration is expressed in seconds

**Table 3:** Relationship between lifetime suicidal behavior severity and TMS measures of cortical excitability and inhibition

TMS measure	Lifetime suicidal behavior severity				
	Correlation coefficient (*r*_s_)	Parameter estimate	Standard error	95% CI for parameter estimate	*p*-value^a^ (*p*_FDR_)
Cortical excitability					
RMT^b^	−0.1058	−0.5160	1.0814	−2.6356 to 1.6036	0.6333 (0.6333)
ICF^c^					
ISI = 10 ms	−0.1788	−0.0655	0.0490	−0.1615 to 0.0305	0.1812 (0.5412)
ISI = 15 ms	−0.1277	−0.0410	0.0485	−0.1361 to 0.0541	0.3986 (0.5412)
ISI = 20 ms	−0.1590	−0.0483	0.0582	−0.1623 to 0.0657	0.4059 (0.5412)
Cortical inhibition					
CSP^d^	−0.2288	−0.0058	0.0032	−0.0121 to 0.0005	0.0723 (0.1446)
SICI^c^					
ISI = 2 ms	−0.2144	−0.0213	0.0268	−0.0737 to 0.0312	0.4268 (0.6402)
ISI = 4 ms	−0.0075	−0.0069	0.0350	−0.0754 to 0.0616	0.8439 (0.8439)
LICI^c^					
ISI = 100 ms	0.4141	0.0792	0.0276	0.0251 to 0.1334	0.0041 (0.0123)
ISI = 150 ms	0.3612	0.0953	0.0304	0.0357 to 0.1549	0.0017 (0.0102)
ISI = 200 ms	0.0205	−0.0266	0.0526	−0.1297 to 0.0766	0.6138 (0.7366)

Note: *p*_FDR_ calculated separately by cortical excitability vs. inhibition. Parameter estimate (from robust regression with MM estimation) was adjusted for age, sex, and CDRS-R total score. Standard error is of the parameter estimate. *r*_s_ = Spearman partial correlation coefficient (which can be interpreted as the effect size estimator).

*RMT* resting motor threshold, *ICF* intracortical facilitation, *ISI* interstimulus interval, *CSP* cortical silent period, *SICI* short-interval intracortical inhibition, *LICI* long-interval intracortical inhibition, *FDR* False Discovery Rate described by Benjamini and Hochberg [32]

^a^Test of the regression relationship between lifetime suicidal behavior severity and each of the TMS outcome measures

^b^RMT is expressed as the percentage of maximal device output

^c^ICF, SICI, and LICI conditioned motor evoked potential (MEP) amplitudes are expressed as ratios to the mean unconditioned MEP amplitudes

^d^CSP duration is expressed in seconds

### Cortical inhibition

**Fig. 2:**
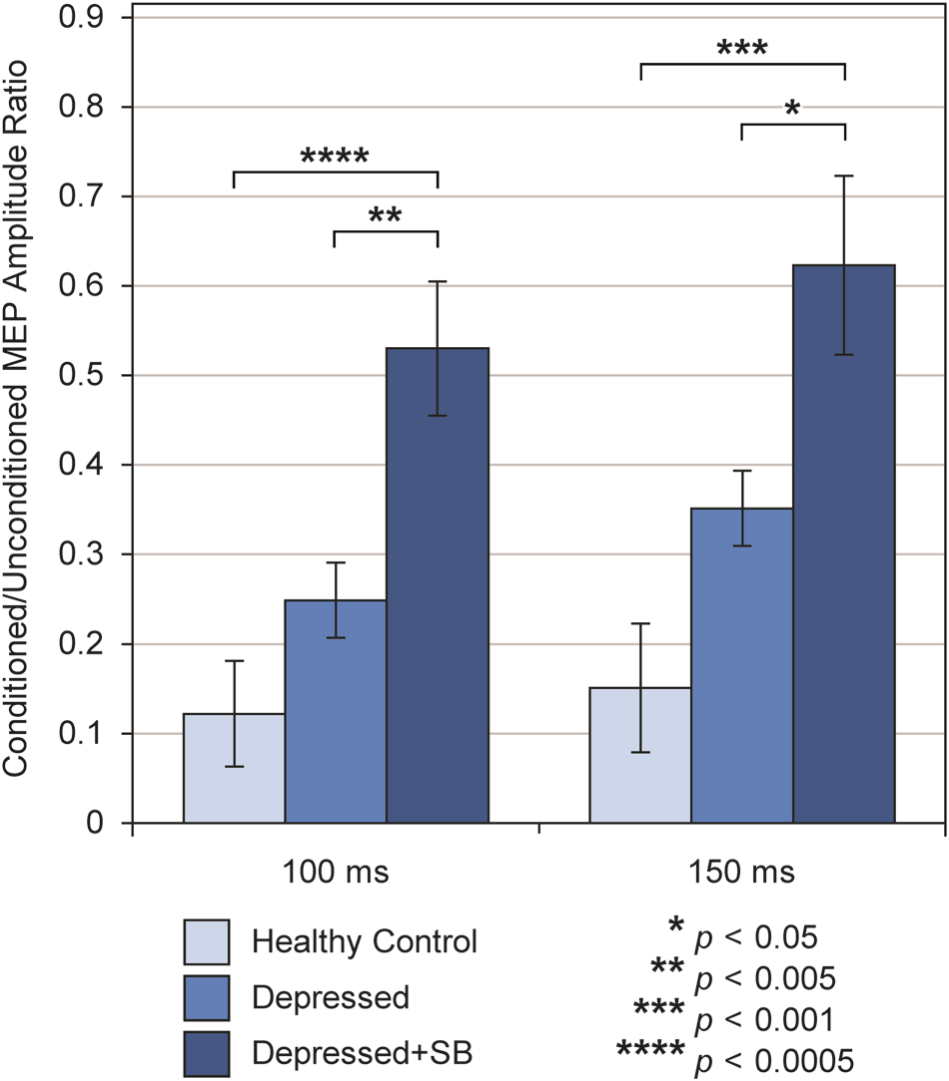
Long-interval intracortical inhibition (LICI) by group. Mean conditioned/unconditioned MEP amplitude ratio values at interstimulus intervals (ISIs) of 100 ms and 150 ms are displayed by group. Error bars represent robust/empirical standard error estimates. Note that higher conditioned/unconditioned MEP amplitude ratio values in the LICI paradigm reflect impairment in cortical inhibition and GABA_B_-mediated inhibitory neurotransmission.

The GLM analysis revealed no significant group main effect for CSP duration (*F*_2,59_ = 2.18, *p* = 0.1216, *p*_FDR_ = 0.2432) or for conditioned/unconditioned MEP amplitude ratio in the SICI paradigm at the 2-ms ISI (*F*_2,68_ = 0.50, *p* = 0.6061, *p*_FDR_ = 0.6061) or 4-ms ISI (*F*_2,67_ = 0.67, *p* = 0.5158, *p*_FDR_ = 0.6061). However, significant group main effects did emerge for conditioned/unconditioned MEP amplitude ratio in the LICI paradigm at 100 ms (*F*_2,55_ = 10.08, *p* = 0.0002, *p*_FDR_ = 0.0012) and 150 ms (*F*_2,54_ = 7.51, *p* = 0.0013, *p*_FDR_ = 0.0039), but not at the 200-ms ISI (*F*_2,56_ = 0.67, *p* = 0.5147, *p*_FDR_ = 0.6061). In post hoc group comparisons (pair-wise contrasts), the pattern of the least squares means showed that the Depressed+SB group demonstrated significantly higher mean conditioned/unconditioned MEP amplitude ratio than the Healthy Control (*t*_55_ = 4.38, *p*_adjusted_ = 0.0002) and Depressed (*t*_55_ = 3.29, *p*_adjusted_ = 0.0049) groups in the 100-ms LICI paradigm, while the Depressed and Healthy Control groups did not differ (*t*_55_ = 1.52, *p*_adjusted_ = 0.2913) (Fig. 2). In the 150-ms LICI paradigm, the pattern of the least squares means showed that the Depressed+SB group demonstrated significantly higher mean conditioned/unconditioned MEP amplitude ratio than the Healthy Control (*t*_54_ = 3.87, *p*_adjusted_ = 0.0009) and Depressed (*t*_54_ = 2.49, *p*_adjusted_ = 0.0418) groups, while the Depressed and Healthy Control groups did not differ (*t*_54_ = 2.17, *p*_adjusted_ = 0.0860) (Fig. 2). Least squares mean and standard error values for TMS measures of cortical inhibition are reported in Table 2. Note that higher conditioned/unconditioned MEP amplitude ratio values in the LICI paradigms reflect impairment in cortical inhibition and GABA_B_-mediated inhibitory neurotransmission.

**Fig. 3:**
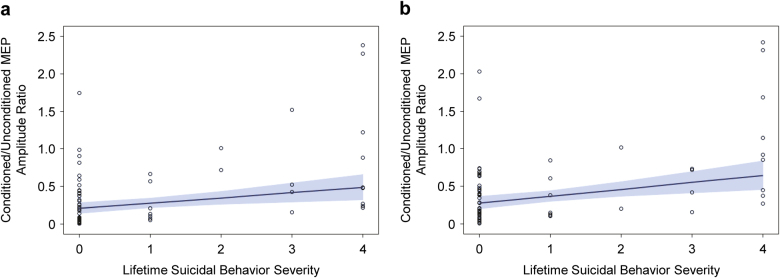
Relationship between severity of lifetime suicidal behavior and LICI. Scatterplots of the conditioned/unconditioned MEP amplitude ratio values in the LICI paradigms (**a** 100-ms ISI; **b** 150-ms ISI) are plotted against lifetime suicidal behavior severity, with fitted robust regression lines and 95% confidence limits, in the overall sample of adolescent participants.

The multiple linear robust regression, while adjusting for age, sex, and CDRS-R total score, revealed a non-significant negative linear relationship between lifetime suicidal behavior severity and CSP (*b̂* = -0.0058, *p* = 0.0723) and between lifetime suicidal behavior severity and conditioned/unconditioned MEP amplitude ratio in the 2-ms (*b̂* = -0.0213, *p* = 0.4268) and 4-ms (*b̂* = -0.0069, *p* = 0.8439) SICI paradigms. However, the robust regression did reveal significant positive linear relationships between severity of lifetime suicidal behavior and conditioned/unconditioned MEP amplitude ratio in the LICI paradigm at the 100-ms ISI (*b̂* = 0.0792, *p* = 0.0041, *p*_FDR_ = 0.0123) and 150-ms ISI (*b̂* = 0.0953, *p* = 0.0017, *p*_FDR_ = 0.0102), but not at the 200-ms ISI (*b̂* = -0.0266, *p* = 0.6138). Robust regression results are reported in Table 3. We also present scatterplots of the conditioned/unconditioned MEP amplitude ratio values against lifetime suicidal behavior severity, with a fitted robust regression line and 95% confidence limits, for the 100-ms (Fig. 3a) and 150-ms (Fig. 3b) LICI paradigms.

**Fig. 4:**
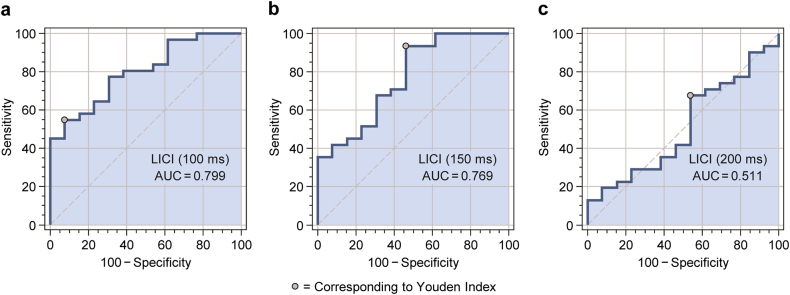
Receiver operating characteristic (ROC) curves for discriminating Depressed vs. Depressed+SB youth in the **a** 100-ms, **b** 150-ms, and **c** 200-ms LICI paradigms.

The ROC analysis determined that the 100-ms LICI paradigm, with a conditioned/unconditioned MEP amplitude ratio cutoff ≤0.2108 (based on the Youden index), best discriminated Depressed youth from Depressed+SB youth (AUC = 0.799, SE = 0.068, 95% binomial exact CI: 0.651–0.905, *Z* = 4.34, *p* = 0.0001, *p*_FDR_ = 0.0003), with 54.84% sensitivity, 92.31% specificity, a PPV of 94.40%, and an NPV of 46.20%. In the 150-ms LICI paradigm, the ROC analysis determined that a conditioned/unconditioned MEP amplitude ratio cutoff ≤0.7003 (based on the Youden index) best discriminated Depressed youth from Depressed+SB youth (AUC = 0.769, SE = 0.081, 95% binomial exact CI: 0.618–0.883, *Z* = 3.32, *p* = 0.0009, *p*_FDR_ = 0.0014), with 93.55% sensitivity, 53.85% specificity, a PPV of 82.90%, and an NPV of 77.80%. The ROC analysis, however, determined that the 200-ms LICI paradigm, with a conditioned/unconditioned MEP amplitude ratio cutoff ≤0.9246 (based on the Youden index), did not significantly discriminate Depressed youth from Depressed+SB youth (AUC = 0.511, SE = 0.097, 95% binomial exact CI: 0.356–0.665, *Z* = 0.11, *p* = 0.9086, *p*_FDR_ = 0.9086, 67.74% sensitivity, 46.15% specificity, a PPV of 75.00%, and an NPV of 37.50%). The ROC curves for the LICI paradigms (100-ms, 150-ms, and 200-ms ISIs) are shown in Fig. 4a–c.

## Discussion

Long-interval intracortical inhibition [33–37] is thought to reflect cortical inhibitory processes mediated by the metabotropic GABA_B_ receptor. This is based on the chronology of the LICI effect, whose range of ISIs corresponds to the timing of inhibitory postsynaptic potentials mediated by the GABA_B_ receptor in motor cortical neurons [36, 38], as well as the higher intensity of the conditioning stimulus necessary for LICI (compared to SICI), which mirrors the higher thresholds required for activation of GABA_B_ receptors relative to GABA_A_ activation thresholds [36]. Additionally, LICI is potentiated by the GABA_B_ receptor agonist baclofen [39] and agents that increase synaptic GABA, such as tiagabine [38] and vigabatrin [40], while benzodiazepines (positive allosteric modulators at the GABA_A_ receptor) do not impact LICI [41, 42].

In our study, there was a significant group effect for LICI at ISIs of 100 ms and 150 ms, with adolescents in the Depressed+SB group exhibiting significantly higher conditioned/unconditioned MEP amplitude ratios (i.e., impaired LICI) than adolescents in either the Healthy Control or the Depressed groups in post hoc comparisons. Although the Depressed group’s mean conditioned/unconditioned MEP amplitude ratio values were intermediate between those of the Healthy Control and Depressed+SB groups, the differences between the Depressed and Healthy Control groups were not significant. This suggests that GABA_B_-mediated inhibitory processes are more disrupted in depressed adolescents with suicidal behavior than in those with depression alone. While the Depressed+SB group had slightly higher mean depression severity than the Depressed group (CDRS-R total scores of 45.88 vs. 41.48), this does not represent a clinically meaningful difference, and furthermore, our analysis included depression severity as a covariate. Thus, the observed differences in LICI between Depressed and Depressed+SB adolescents are unlikely to represent the effect of more severe depressive symptoms in the adolescents with histories of suicidal behavior. Impairment in LICI may instead be related uniquely to history of suicidal behavior in this depressed adolescent sample. Additionally, in the multiple linear robust regression analysis for the 100-ms and 150-ms LICI paradigms, there were significant positive linear relationships between the ordinal lifetime suicidal behavior severity variable and the conditioned/unconditioned MEP amplitude ratios. This raises the question of whether increasingly severe suicidal behavior corresponds to greater deficits in cortical inhibition. Furthermore, the ROC analysis suggested that LICI could discriminate the presence and absence of a lifetime history of suicidal behavior among depressed youth, with the 150-ms paradigm exhibiting superior sensitivity and the 100-ms paradigm demonstrating superior specificity. The absence of group differences in LICI at the 200-ms ISI, of a significant relationship between suicidal behavior severity and LICI in the 200-ms regression, and of better-than-chance discriminating ability for 200-ms LICI in the ROC analysis may be related to late disinhibition effects [37, 43, 44] that overlap with and persist beyond LICI, representing a gradual transition from net inhibition to net facilitation that occurs in cortical networks following magnetic stimulation.

The Depressed+SB group exhibited the shortest mean CSP duration, while Healthy Controls had the longest mean CSP; however, the group effect failed to reach the threshold for significance. Like LICI, the CSP is posited to have GABA_B_-mediated mechanisms [36, 45], but the two effects may involve distinct populations of cortical neurons [46] or be modulated by different cortico-cortical inputs [47]. Additionally, other work has demonstrated the effects of GABA_A_ receptors on the CSP under certain conditions [48]. Notably, SICI (a putative index of GABA_A_-mediated inhibition) did not differ between groups in our study.

Our findings suggest that cortical inhibition mediated by the GABA_B_ receptor, rather than the GABA_A_ receptor, may be altered in adolescents with suicidal behavior. Interestingly, this stands somewhat in contrast to the adult literature, which is notable for a number of studies that implicate the GABA_A_ receptor in suicide and a paucity of evidence for GABA_B_ involvement [11–14, 18]. One potential explanation is that the composition and function of cortical GABAergic inhibitory systems is dynamic throughout neurodevelopment. In early life, GABA_A_ receptor activation results in depolarization, later transitioning to its mature inhibitory function [49, 50]. This may be related in part to differential expression of the various GABA_A_ receptor subunits, which shifts substantially from infancy through adulthood [51, 52], as well as age-related changes in the expression of cotransporters affecting the receptor’s function [50, 52]. GABA_B_ receptor functions also undergo developmental changes; whereas GABA_B_-mediated presynaptic inhibition is present at birth, postsynaptic inhibition is absent in early development [49, 53]. Additionally, GABA receptor density continues to change into early adulthood, although at different rates in different brain regions, with the cortex maturing at a later age than subcortical structures [54]. These shifts in GABA receptor function and distribution, interacting with the simultaneous development of glutamatergic systems [49, 52], results in a highly dynamic cortical excitatory–inhibitory balance during childhood and adolescence, with mature GABAergic inhibition arising gradually and relatively late in brain development [50, 52]. Another important caveat in comparing our findings to previous adult research is that the majority of adult studies involved the use of gene- or receptor-specific methodologies to examine postmortem brain tissue of suicide victims, whereas the TMS measures utilized in our study measure excitatory and inhibitory physiologic functions in vivo.

Cortical inhibitory deficits in suicidal individuals may have important implications for the development of treatments to reduce suicide risk. Sun et al. [55] examined TMS-electroencephalographic measures of cortical inhibition (LICI and N100) in the left dorsolateral prefrontal cortex (L-DLPFC) and motor cortex in adults undergoing magnetic seizure therapy (MST) for treatment-resistant depression. The combination of LICI and N100 in the L-DLPFC predicted remission of suicidal ideation with 89% accuracy, 90% sensitivity, and 89% specificity, with better frontal inhibitory functioning at baseline corresponding to greater likelihood of remission of suicidal ideation with MST [55]. The authors postulated that integrity of inhibitory interneuronal circuits in the frontal cortex mediates the therapeutic response to MST. In addition to serving as a potential predictor of response, cortical inhibition may itself be a target for intervention in suicidal patients. Neuromodulatory techniques, including repetitive TMS (rTMS), are thought to exert their effects through GABAergic mechanisms. Cortical inhibition improves after high-frequency rTMS [56], particularly in individuals with reduced inhibition prior to stimulation [57]. If cortical inhibitory processes are indeed central to the pathophysiology of suicidal behavior, therapeutic interventions that modulate GABAergic networks and improve inhibitory functioning may have great value in future treatment of suicidal individuals.

The current study has some notable limitations. First, the overall sample, while comparable to or larger than those in previous TMS neurophysiology studies, was modest in size, particularly the Depressed+SB group. Future studies, especially those seeking to establish the prognostic utility of cortical inhibition in assessing suicide risk, will require larger numbers of young participants with suicidal behavior. Second, the three groups differed in age (*p* =  0.005, *p*_FDR_ = 0.015), with the Healthy Control group’s mean age (14.20 ± 1.76) being younger than those of the Depressed (15.70 ± 1.88) or Depressed+SB (15.88 ± 1.57) groups. However, prior work [58] found that LICI is inversely correlated with age in both healthy control and depressed youth; thus, it would be expected that a healthy control group more closely matched in age to the depressed groups might show even greater inhibition of the conditioned MEP (and therefore greater differentiation from the Depressed and Depressed+SB groups). Participants in our study ranged from 12 to 20 years in age, complicating the assessment of depressive symptoms across such a broad span. While the CDRS-R has been utilized in numerous prior studies of depression and suicidality in adolescents (e.g., refs. [59–63]), it was designed originally for younger populations, and future studies that include young adults should include measures that are more developmentally appropriate to this age group. Furthermore, a large majority of the adolescents with histories of suicidal behavior in our sample were treated pharmacologically with selective serotonin reuptake inhibitors or serotonin-norepinephrine reuptake inhibitors, as were more than half of the depressed adolescents without suicidal behavior. While this is representative of many adolescents encountered in clinical practice (particularly those whose suicidal behavior has come to clinical attention), it does pose a potential confound, as antidepressant medications have been shown to potentiate some TMS measures of cortical inhibition such as CSP and SICI [64–66]. However, to our knowledge, the effect of antidepressant medications on LICI has not been examined. Although no participants in the current study reported use of benzodiazepines or other medications with direct GABAergic mechanisms, future investigators may wish to confirm the absence of confounding substances with urine or serum assays.

One major caveat in the interpretation of our results is the fact that the Depressed+SB group consisted of depressed adolescents with historical suicidal behavior; none had ongoing suicidal intent or preparatory behavior at the time of participation. This increases the possibility of recall bias as well as the likelihood of both type I and type II errors. While the inclusion of historically suicidal participants was necessary for pragmatic and ethical reasons, the present study did not assess whether disturbances in cortical excitability or inhibition are present in adolescents who are in the midst of acute suicidal crises. It is possible that acutely suicidal adolescents have similar patterns of impaired cortical inhibition or even more extreme LICI deficits; however, it is equally possible that acute suicidal crises have fundamentally different underlying neurobiology. Markers of disrupted excitatory–inhibitory balance have been shown to improve with time after suicide attempts [19], suggesting that neurobiological correlates of suicidality are at least partially dynamic. Studies that directly compare persons with active or very recent suicidal behavior with persons whose suicidal behavior is more distant, as well as prospective studies that repeat neurobiological assessments over time as suicidal risk changes, are necessary to answer this important “state-versus-trait” question, which has significant implications for the use of cortical inhibitory measures in assessing suicide risk. Additionally, all adolescents with histories of suicidal behavior included in our sample had diagnoses of unipolar depressive disorders. Much of the literature implicating GABA and glutamate in suicide is derived from unipolar depressed populations, although there are exceptions [11, 12, 14, 19, 20]. Despite our findings that indicate substantially greater cortical inhibitory impairment among adolescents with histories of suicidal behavior, caution should be exercised in generalizing this finding to adolescents with suicidal behavior in the context of other psychopathology. Further research is needed in additional adolescent populations with high rates of suicidal behavior (e.g., bipolar disorder, schizophrenia, obsessive-compulsive disorder, substance use disorders, and childhood adversity) to determine whether similar patterns of impaired cortical inhibition are present in suicidality across diagnostic groups. While the groups in our study were defined by a categorical diagnosis (depression) and the presence or absence of suicidal behavior history, the heterogeneity of depressive symptoms and diversity of suicidal histories are problematic when investigating the neurobiological correlates of such complex syndromes and behaviors. The use of transdiagnostic dimensional measures, as advocated by the National Institute of Mental Health’s Research Domain Criteria initiative [67, 68], may yield more clinically relevant findings in suicide risk research than prior work on sociodemographic and diagnostic risk factors [69, 70]. Future studies should consider assessing dimensional constructs with particular relevance to both suicidal behavior and GABAergic neurotransmission, such as aggression and frustrative nonreward, anhedonia, fear and anxiety, impulsivity, and irritability, which may be important independent factors or may mediate suicidal behavior through interactions with neural risk factors, such as impaired cortical inhibition. Finally, the predictive utility of any single risk factor for suicidal behavior is likely to be limited, suggesting the need for combination or algorithmic approaches to risk assessment [6]. The accuracy and sensitivity of cortical inhibition in predicting a suicide-related outcome were improved by using two measures rather than one [55], and future investigations should explore combinations of promising biomarkers in order to optimize clinical usefulness.

To our knowledge, the current study represents the first examination of TMS-measured cortical excitability and inhibition in adolescents with suicidal behavior. The finding of impaired cortical inhibition, as indexed by LICI, suggests that GABAergic neurotransmission has a role in the pathophysiology of suicidal behavior in this population, possibly distinct from the effect of the depressive illness. Cortical inhibition is a promising target for further research into risk assessment and interventions for suicidal behavior in adolescents.

## Electronic supplementary material

Supplmentary Materials
