# Supplementary material for: Cortical inhibitory markers of lifetime suicidal behavior in depressed adolescents
Source: Neuropsychopharmacology. 2018 Mar 14;43(9):1822–31. doi: 10.1038/s41386-018-0040-x (PMC6046050; doi:10.1038/s41386-018-0040-x)
Supplement: Supplementary file 1 — Supplmentary Materials [file 41386_2018_40_MOESM1_ESM.pdf]

Lewis CP, Nakonezny PA, Blacker CJ, Vande Voort JL, Port JD, Worrell GA, Jo HJ, Daskalakis ZJ, Croarkin PE. Cortical Inhibitory Markers of Lifetime Suicidal Behavior in Depressed Adolescents.

SUPPLEMENTARY MATERIALS AND METHODS

Statistical Analysis

The primary analysis was a fixed-effects general linear model (GLM), which was used to examine the group main effect (Healthy Controls vs. Depressed vs. Depressed+SB) on each of the TMS outcome measures of cortical inhibition (CSP, SICI, LICI) and excitability (RMT, ICF). A separate GLM was conducted on each TMS outcome. Depression severity (CDRS-R total score), age, and sex were included as covariates in the model. Restricted maximum likelihood estimation and robust standard errors (sandwich HC3 first order residual estimator) along with Type 3 tests of fixed effects were implemented. Group least squares means of the TMS estimates were compared for a total of three *post hoc* pair-wise contrasts, with *p*-values adjusted for multiple comparisons using the Tukey-Kramer method. A weight statement was included in the model to account for (and down-weight) potential outliers. First, weights were estimated using Tukey's Bisquare weight function from MM estimation in SAS' PROC ROBUSTREG (with the regression of each TMS outcome on age, sex, CDRS-R score, and group; same model specification as explicated above). Then, with respective weights for each observation, the aforementioned fixed-effects general linear model was fit using the procedures of PROC GLIMMIX in SAS. All participants were included in the weighted analysis, but weighted according to the Tukey Bisquare function (Rousseeuw and Leroy, 1987), an iteratively reweighted measure of central tendency (with weights ranging from 0 to 1). The bisquare

depends on the calculated weights and the weights depend on the bisquare. The M-estimator becomes the new measure of central tendency for each subsequent iteration. Thus, as an iteratively reweighted measure of central tendency, the weighted analysis (using the Tukey Bisquare function) gives less weight as the distance of the observed data point increases away from the M-estimator (for distances larger than 4.685, Tukey's constant cutpoint, the weight equals zero) and more weight as the distance between the observed data point and the M-estimator becomes smaller (but ≤ 4.685; the weight approaches 1 as the distance approaches 0).

In a secondary analysis, multiple linear robust regression (with MM estimation) and the Spearman partial correlation coefficient (r_s) were used to examine the relationship between severity of lifetime suicidal behavior and each TMS outcome measure, while adjusting for age, sex, and CDRS-R total score. The estimated slope from the linear regression model indicates the mean change in each TMS measure per one-scale unit increase in severity of suicidality, while the sample correlation coefficient indicates direction and strength of the linear relationship between severity of suicidality and each TMS measure. The Spearman partial correlation coefficient can also be interpreted as the effect size estimator in evaluating the magnitude of the relationship between severity of suicidal behavior and each TMS measure.

SUPPLEMENTARY REFERENCES

Rousseeuw PJ, Leroy AM (1987). *Robust regression and outlier detection*. John Wiley & Sons: New York.

Table S1. Psychotropic Medications of Depressed Participants at Time of TMS Testing

Participant	Group	Medication(s) and Total Daily Dose
Study 2-001	Depressed	none
Study 2-002	Depressed	fluoxetine 40 mg
Study 2-003	Depressed	none
Study 2-004	Depressed	fluoxetine 20 mg
Study 2-005	Depressed	fluoxetine 30 mg
Study 2-006	Depressed	escitalopram 10 mg
Study 2-007	Depressed	none
Study 2-008	Depressed	none
Study 2-009	Depressed	none
Study 2-010	Depressed	none
Study 2-011	Depressed	none
Study 2-012	Depressed+SB	escitalopram 10 mg
Study 2-013	Depressed+SB	fluoxetine 60 mg, trazodone 75 mg, methylphenidate 60 mg*
Study 2-014	Depressed	escitalopram 20 mg
Study 2-015	Depressed	escitalopram 10 mg
Study 2-016	Depressed	fluoxetine 20 mg
Study 2-017	Depressed+SB	escitalopram 10 mg
Study 2-018	Depressed+SB	fluoxetine 10 mg
Study 2-019	Depressed	sertraline 100 mg
Study 2-020	Depressed	methylphenidate 18 mg*
Study 2-021	Depressed	sertraline 200 mg, aripiprazole 2 mg
Study 2-022	Depressed	fluoxetine 20 mg
Study 3-001	Depressed+SB	none
Study 3-002	Depressed	fluoxetine 40 mg, lisdexamfetamine 70 mg*, amphetamine-dextroamphetamine 20 mg*
Study 3-003	Depressed+SB	fluoxetine 30 mg
Study 3-004	Depressed+SB	sertraline 150 mg, aripiprazole 2 mg
Study 3-005	Depressed+SB	fluoxetine 40 mg
Study 3-006	Depressed	none
Study 3-007	Depressed	none
Study 3-008	Depressed	fluoxetine 40 mg, trazodone 50 mg
Study 3-009	Depressed	escitalopram 20 mg, topiramate 150 mg
Study 3-010	Depressed+SB	sertraline 50 mg
Study 3-011	Depressed+SB	none
Study 3-012	Depressed	none
Study 3-013	Depressed	none
Study 3-014	Depressed	none
Study 3-015	Depressed	none
Study 3-016	Depressed+SB	fluoxetine 30 mg, methylphenidate 54 mg*
Study 3-017	Depressed+SB	fluoxetine 20 mg, amphetamine-dextroamphetamine 30 mg*
Study 3-018	Depressed	fluoxetine 20 mg

Study 3-019	Depressed+SB	none
Study 3-020	Depressed	fluoxetine 30 mg
Study 3-021	Depressed+SB	none
Study 3-022	Depressed	duloxetine 120 mg
Study 3-023	Depressed	none
Study 3-024	Depressed	venlafaxine 150 mg
Study 3-025	Depressed+SB	fluoxetine 20 mg
Study 3-026	Depressed	none
Study 3-027	Depressed+SB	none
Study 3-028	Depressed	none
Study 3-029	Depressed	none
Study 3-030	Depressed+SB	fluoxetine 30 mg
Study 3-031	Depressed	amphetamine-dextroamphetamine 40 mg*
Study 3-032	Depressed	fluoxetine 20 mg

* Stimulant medications held on day of TMS testing.
